# Assessment of non-progressive dysarthria: practice and attitude of speech and language therapists in Lebanon

**DOI:** 10.1186/s12883-021-02484-2

**Published:** 2021-11-17

**Authors:** Marwa Summaka, Hayat Harati, Salem Hannoun, Hiba Zein, Nour Koubaisy, Youssef Fares, Zeina Nasser

**Affiliations:** 1 Doctoral School of Sciences and Technology, Lebanese University, Hadath, Lebanon; 2 Faculty of Medical Sciences, Neuroscience Research Center, Lebanese University, Hadath, Lebanon; 3 Medical Imaging Sciences Program, Division of Health Professions, Faculty of Health Sciences, American University of Beirut, Beirut, Lebanon; 4 Department of Rehabilitation, Health, Rehabilitation, Integration and Research Center (HRIR), Beirut, Lebanon

**Keywords:** Non-progressive dysarthria, Assessment, Speech and language therapy, Survey, ICF, Lebanon

## Abstract

**Background:**

Non-progressive dysarthria is an acquired motor speech disorder resulting from neurological diseases such as stroke and traumatic brain injury. The evidence base for the assessment of non-progressive dysarthria remains limited with professional practices relying mainly on therapists’ clinical experience. Limited information on the assessment practices of Lebanese speech and language therapists (SLTs) is available. Such information is crucial for the development of adequate therapy services for clients with non-progressive dysarthria. This study aims to explore the assessment practices and attitudes of Lebanese SLTs working with adults with non-progressive dysarthria and to investigate their adherence to the framework of the World Health Organization’s International Classification of Functioning, Disability and Health (ICF).

**Methods:**

A cross-sectional study was conducted in Lebanon between March and May 2021. Data was collected through an online survey that included information on socio-demographic characteristics, practices, and attitudes of SLTs who assess adults with non-progressive dysarthria.

**Results:**

A total of 50 Lebanese SLTs responded to the survey. The majority of SLTs (78%) assessed clients with non-progressive dysarthria across all ICF domains. SLTs reported dissatisfaction with the available assessment tools (64%) and reliance on informal tools (84%). In addition, 68% of the SLTs suggested the crucial need for the development of Arabic formal assessments that can quantitatively evaluate dysarthria and determine severity. The survey also showed that the respondents demonstrated a preference for the use of impairment-based tools.

**Conclusion:**

It can be concluded that the assessment practices of Lebanese SLTs, generally, follow the international trend and the recommended professional guidelines. Further research initiatives should be held to develop Arabic formal assessment tools for non-progressive dysarthria.

**Supplementary Information:**

The online version contains supplementary material available at 10.1186/s12883-021-02484-2.

## Background

Non-progressive dysarthria is defined as a neurologic motor speech disorder that is induced by stroke, traumatic brain injury, or other non-progressive neurological conditions [1]. It can affect communication due to poor coordination of the muscles involved in respiration, phonation, articulation, and prosody [1, 2]. Speakers with dysarthria mostly manifest reduced intelligibility, slurred speech, and altered prosody that places perceived efforts on the speaker during conversations [1, 2]. Non-progressive dysarthria can occur in a wide severity range; however, it doesn’t need to be severe to impact people’s interaction and social participation [3–6]. Regarding its prevalence, dysarthria affects 20–42% of stroke survivors [7, 8], as well as, 10–60% of traumatic brain injury survivors [9]. Despite its frequency, non-progressive dysarthria receives less attention in literature when compared to progressive dysarthria [1, 3, 4], with a considerable focus on neurodegenerative diseases [10–14]. Progressive dysarthria represents a common consequence of neurodegenerative diseases, where the motor speech disorder worsens as the disease progresses [15]. Both, progressive and non-progressive dysarthria follow distinct care pathways.

Assessment in dysarthria focuses on determining the existence, pattern, and severity of the disorder as well as its effect on the speech subsystems to establish the appropriate rehabilitation plan [16, 17]. The assessment also involves investigating the influence of the physiological impairment on people’s daily activities, psychosocial functions, and health-related quality of life [16, 18]. The World Health Organization’s International Classification of Functioning, Disability and Health (ICF) [19] recommended a holistic framework in understanding and assessing dysarthria [20]. This approach identifies disability as comprising impairment in body structures and functions (e.g., reduced strength and speed of speech musculature), activity restrictions (e.g., reduced intelligibility), and participation constraints (e.g., limitations in performance at different settings such as work, university, social events, etc.).

There is a significant gap in the literature concerning the practices of speech and language therapists (SLTs) in people with non-progressive dysarthria and whether their practices reflect the framework suggested by the ICF. Till now, only three studies have been conducted for this aim in the Republic of Ireland (ROI), United Kingdom (UK), and Australia [16, 17, 21]. The studies conducted in the ROI and UK reported the assessment and management practices of SLTs working with people with non-progressive dysarthria and post-stroke dysarthria respectively [16, 21]. Results indicated that SLTs relied mainly on informal assessments, with extremely limited use of instrumental assessments. SLTs’ practices extended beyond the impairment level and they indicated providing high value to the assessment and outcomes of activity limitations and participation restrictions [16, 21]. Additionally, ROI SLTs related several barriers to assessment in non-progressive dysarthria including reduced availability of resources and trainings, limitations of some available assessment tools, and the presence of comorbidities in clients [16].

The most recent study on the assessment practices of SLTs in the field of non-progressive dysarthria was performed in Australia including 56 respondents [17]. Australian SLTs favored informal assessments over standardized or published tools, with rare use of instrumental tools. Reported barriers to the implementation of formal assessments included time constraints, lack of required resources, and unsatisfactory sensitivity of tools. Their assessment practices focused primarily on the impairment and activity limitations and less on the participation restrictions.

Given this background, emerged results demonstrated variability in assessment practices provided for adults with non-progressive dysarthria across the mentioned countries. To date, there have been no published studies about the assessment practices of Lebanese SLTs in the field of non-progressive dysarthria. It is well known that non-progressive dysarthria is a common sequel of stroke [7, 22, 23] and traumatic brain injury (TBI) [24], which are notably prevalent in Lebanon. So that, there is a relatively higher prevalence of stroke in Lebanon when compared with other developing countries [25]. Indeed, the rates of TBI are increased in Lebanon, with war and blast-induced TBIs being the main cause, which is possibly related to the war-inflicted geographical region where Lebanon resides [26]. Taken together, non-progressive dysarthria is considered an important area of investigation in Lebanon that suffers from major flaws pertaining to the lack of standardized assessments or outcome measures. To develop formal assessment tools and enhance provided services for adults with non-progressive dysarthria in Lebanon, adequate information about the practices of Lebanese SLTs should be available. Accordingly, the primary aims of the study are to (1) investigate the current assessment practices and attitudes of Lebanese SLTs working with adults having non-progressive dysarthria, (2) identify the challenges faced by SLTs during the assessment process, and (3) inspect the accordance of the findings with the ICF domains.

## Methods

### Study design and participants

This is a cross-sectional study conducted in Lebanon from March to May 2021. Lebanese SLTs were eligible to participate in this study if they work in Lebanon with adults with acquired non-progressive dysarthria. The current study included 50 participants recruited via an online questionnaire survey.

### Instrumentation

The questionnaire of the survey was developed by the authors (Additional file 1) after reviewing the relevant published literature [16, 17, 21]. The questionnaire was designed in the English language to cover important aspects in the assessment and outcome measure practices of Lebanese SLTs working with adults with non-progressive dysarthria. An independent committee consisting of five experts with expertise in speech therapy and specifically neurorehabilitation reviewed in-depth the first draft of the questionnaire. They assessed the relevance of the items regarding the presentation of dysarthria and clinical application. A consensus was reached, and a preliminary final version of the questionnaire was developed. All questions were required to be answered before the final submission of responses to avoid missing data. The online survey included the following sections:

1. Demographics: it included the baseline characteristics of participants such as age, gender, marital status, highest educational achievement, years of experience, and primary workplace.
2. General information about caseload: it included information related to the portion of caseload spent with adults with non-progressive dysarthria, the underlying medical conditions resulting in non-progressive dysarthria, and the sources of patients’ referrals.
3. Practices section: it included 2 dimensions (assessments and outcome measures) with a total of 30 questions measuring the assessment and outcome measure practices of Lebanese SLTs. The questions were about components of speech productions being assessed, frequency of applying informal assessments, implementation of specific descriptive assessments, frequency of using formal assessment tools, utilizing instrumental assessments, frequency of assessing non-progressive dysarthria beyond the impairment level, reliance on the Mayo classification system and frequency of utilizing outcome measures. Respondents were required to answer in terms of Likert-type responses of “never”, “some of the time”, “most of the time, and “always”. In addition, the practices section encompassed 3 short questions allowing SLTs to specify formal, informal, and instrumental assessments and 6 open-ended questions about the barriers faced by Lebanese SLTs during the process of assessment.
4. Attitude section: this section included 24 questions investigating the attitude of SLTs towards statements regarding the assessment of adults with non-progressive dysarthria. Participants had to state their level of agreement with statements related to the assessment procedure, implementation of specific tasks, reasons behind the assessment, and satisfaction level toward available assessment tools. Answers were expressed in terms of a Likert scale: “agree”, “neutral” and “disagree”.

### Pilot testing

To make sure that the questionnaire was clear and comprehensible, it was piloted on 4 Lebanese SLTs experienced in the field acquired neurorehabilitation. The survey was then revised and modified following the amendments of the participants regarding the ambiguity of a few questions/statements. On average, the survey required 12 min to be completed.

### Data collection

Enrolled SLTs were reached out via social media and advertisements sent via e-mail by the official platforms of the Lebanese Speech therapists associations (Association Libanaise des Orthophonistes-ALO) and the Superior Institute of Speech therapy at the Saint Joseph University (Institut Supérieur d’orthophonie-ISO-USJ). A reminder e-mail was sent again to the SLTs 3 weeks later. The online survey was held using google forms. An invitation letter associated with a link to the survey was prepared and sent to the participants. The invitation letter includes information about the purpose of the study and requesting the voluntary participation of SLTs. The survey was sent to approximately 400 Lebanese SLTs. According to the ALO, around 500 SLTs are practicing in Lebanon, however, the exact number of SLTs working with non-progressive dysarthria is unknown. Therefore, the response rate couldn’t be estimated. The methods were performed in accordance with the Declaration of Helsinki [27].

### Data analysis

The data analysis was carried out using IBM SPSS software (Statistical Package for Social Sciences), version 22.0. to be analyzed. Descriptive statistics were determined using means and standard deviations (SD) for continuous variables, and frequency with percentages for categorical variables. Some variables were collapsed for analytical purposes. The Chi-square test was used to evaluate any associations between variables. As for qualitative data, they were analyzed manually and categorized based on key themes. The qualitative analysis of respondent’s comments was performed by two researchers. The statistical tests used were two-sided, with the significance level set at 0.05.

## Results

### Characteristics of the study respondents

Table 1 depicts the baseline characteristics of study respondents. A total of 50 Lebanese SLTs participated in this study among them 96% were females. Their ages ranged between 22 and 43, with a mean of 28.64 ± 5.33. The majority possessed a postgraduate master’s degree (58%) and 40% had a bachelor’s degree in speech and language therapy. Years of experience of respondents working with individuals with non-progressive dysarthria ranged between 1 and 20 years, with a mean of 5.78 ± 4.52 years. The highest proportion of respondents reported that they spent less than or about 25% of their caseload working with individuals with non-progressive dysarthria (78%). Most respondents provided speech and language therapy services at the client’s home (44%) and 26% worked in rehabilitation centers. Stroke was the primarily reported etiology of non-progressive dysarthria (62%), followed by traumatic brain injury (26%). Referral sources of clients were from medical doctors (36%), therapists from other specialties (30%), and family decisions (34%).

**Table 1:** Baseline characteristics of study respondents

	Frequency (n)	Percentage (%)
*Gender*		
Male	2	4
Female	48	96
*Marital status*		
Single	19	38
Engaged	7	14
Married	24	48
*Highest level of education in speech therapy*		
Bachelor degree	20	40
Postgraduate Masters degree	29	58
Doctor of Philosophy (Ph.D.)	1	2
*Years of experience*		
*1–5*	28	56
*6–10*	15	30
*More than 10*	7	14
*Primary workplace*		
Acute general hospital	5	10
Client’s home	22	44
Outpatient hospital	5	10
Post-acute/rehabilitation-general hospital/inpatient	3	6
Rehabilitation center	13	26
Telepractice	2	4
*Portion of the current caseload*		
Less than or about 25%	39	78
Less than or about 50%	11	22
*Primary etiologies of non-progressive dysarthria*		
Aneurysm	1	2
Brain cancer	1	2
Cerebral palsy	3	6
Encephalitis	1	2
Stroke	31	62
Traumatic brain injury	13	26
*Referral sources*		
Client’s/family decision	17	34
Medical doctors	18	36
Therapists from different rehabilitative fields	15	30
	Mean ± SD^a^	
*Age*	28.64 ± 5.33	

^a^ Standard deviation

For analytical purposes, years of experience were categorized into “≤5 years” and “> 5 years” and the primary workplace responses were grouped into “hospital-based SLTs” and “community-based SLTs”. There were no significant associations between the use of informal assessments, according to years of experience (X^2^ = 3.84, *p* = 0.15) or clinical workplace (X^2^ = 6.26, *p* = 0.084). No significant link was found between respondents’ perceived importance of assessing functional communication in adults with non-progressive dysarthria and years of experience (X^2^ = 4.14, *p* = 0.126) or clinical workplace (X^2^ = 2.91, *p* = 0.234). Furthermore, there was no significant difference between medical-based SLTs and community-based SLTs in terms of assessing the ICF domains beyond the “impairment” level (X^2^ = 0.84, *p* = 0.64). Therefore, a whole-cohort analysis was carried out.

### Lebanese SLTs’ assessment practices for adults with non-progressive dysarthria

#### Formal assessments

Most of the SLTs reported that they do not routinely use formal assessments (86%), with 50% indicating never using them. On the other hand, 14% of SLTs noted that they use formal assessments “most of the time”. Two respondents (4%) specified using the “batterie d’évaluation clinique de la dysarthrie (BECD)” [28], 6% (*n* = 3) indicated using the Frenchay Dysarthria Assessment (FDA) [29, 30], and 2% (*n* = 1) reported using the Radboud Dysarthria Assessment (RDA) [31]. In addition, 10% (*n* = 5) stated using the Lebanese Arabic version of The Voice Handicap Index-10 (VHI-10 lb) [32]. Regarding the ICF domains corresponding to the reported assessments, all of them are measures of “impairment” except for the VHI-10 lb. which is a measure of “activity limitation”.

#### Informal assessments

The majority of respondents (84%) stated that they “always” or “most of the time” implement informal assessments. Table 2 represents the reported frequency of use of informal descriptive assessments for non-progressive dysarthria, along with the corresponding ICF domains. 94% of the respondents reported that they “always” or “most of the time” perform an oral motor examination in the assessment of non-progressive dysarthria, and no one reported “never” using an oral motor examination. Respondents (90%) also indicated “always” or “most of the time” using informal articulation rating and informal speech rating for adults with non-progressive dysarthria. Impairment measures were the most frequently used ones followed by the activity limitation measures. In addition, 48% of SLTs indicated that they routinely use the Mayo classification system (e.g., flaccid, spastic, unilateral upper motor neuron, etc.) for describing the type of dysarthria, while 52% didn’t routinely use it.

**Table 2:** The reported frequency of use of informal descriptive assessment for non-progressive dysarthria (*n* = 50)

Descriptive assessment	ICF^a^ domain	Always		Most of the time		Some of the time		Never	
		***n***	%	***n***	%	***n***	%	***n***	%
Oral facial examination	**I**	30	60	17	34	3	6	0	0
Informal articulation rating	**I**	28	56	17	34	5	10	0	0
Informal speech rating	**AL**	26	52	19	38	5	10	0	0
Informal intelligibility testing	**AL**	29	58	14	28	7	14	0	0
Conversation/interaction rating	**AL**	27	54	16	32	6	12	1	2
Observation of connected speech in reading	**AL**	20	40	17	34	13	26	0	0
Informal rating scales of speech function (e.g. breath support and phonation)	**I**	18	36	21	42	7	14	4	8
Client’s/significant others’ ratings of social participation	**PR**	13	26	21	42	11	22	5	10
Informal ratings of conversational partner interaction skills	**AL**	14	28	14	28	19	38	3	6

^a^ International Classification of Functioning, Disability and Health domains: *I* Impairment; *AL* Activity Limitations; *PR* Participation Restrictions

#### Components of speech production

Also, respondents reported the frequency of evaluating specific components of speech production (respiration, prosody, resonance, articulation, and phonation) in the assessment of non-progressive dysarthria. Articulation was the most frequently assessed component of speech production; so that it was assessed “always” or “most of the time” by 98% of respondents. Phonation was the second frequently assessed component of speech production, with up to 94% of respondents reportedly evaluating it “always” or “most of the time”. Respiration was also reported to be frequently evaluated in adults with non-progressive dysarthria; 90% of respondents “always” or “most of the time” evaluated respiration. No one of the respondents reported “never” assessing articulation, phonation, or respiration in adults with non-progressive dysarthria. Prosody and resonance were “always” or “most of the time” assessed by 70 and 62% of respondents respectively.

#### Activities and participation domains of assessment

Moreover, SLTs provided information on the frequency of assessing activities and participation domains of the ICF in adults with non-progressive dysarthria (Table 3). The psychosocial impact of dysarthria was “always” or “most of the time” evaluated by 82% of SLTs, followed by the environmental barriers to communication. Depression, anxiety, and attitudinal barriers to communication were the less frequently assessed domains.

**Table 3:** The reported frequency of assessing activities and participation domains in non-progressive dysarthria (*n* = 50)

Area of assessment	Always		Most of the time		Some of the time		Never	
	***n***	%	***n***	%	***n***	%	***n***	%
Communication effectiveness beyond the clinical setting	24	48	15	30	10	20	1	2
Environmental barriers to communication	21	42	19	38	8	16	2	4
Attitudinal barriers to communication	18	36	19	38	11	22	2	4
Depression/anxiety	16	32	21	42	11	22	2	4
Quality of Life	22	44	17	34	10	20	1	2
Psychosocial impact of dysarthria	20	40	21	42	9	18	0	0

#### Instrumental assessment

Respondents reported using instrumentation less frequently in the assessment of adults with non-progressive dysarthria. Most of the SLTs stated “never” using the different proposed instrumental assessments. Also, the majority of respondents (98%) reported “never” using Computerized Speech Lab. 96% of SLTs “never” used Visispeech and Visipitch in the assessment of non-progressive dysarthria. The reported frequency of using Electroglottograph and Analysis of Dysphonia in Speech and Voice was also reduced, so that, 94% “never” used both instrumentations. Almost half of the respondents (58%) indicated “never” using acoustic analysis software packages (e.g., Praat, lingWAVES), while (22%) reported using them “some of the time” and (20%) “most of the time”.

#### Outcome measures

Most of the respondents (76%) reported that they “always” or “most of the time” implement outcome measures, while 24% indicated using them “some of the time”. In terms of the outcome measures selected, 70% of SLTs repeated informal assessments, and 30% implemented goal attainment scaling. SLTs highlighted several purposes behind implementing outcome measures including, evaluating the client’s progress, assessing the effectiveness of intervention goals, collecting data for research purposes, and motivating the clients through comparison with the baseline assessment. Other SLTs commented that they do not implement outcome measures due to time constraints, unexpected discharge of inpatients, and lack of available quantitative measures. Furthermore, most of the SLTs (66%) reported being satisfied with the available outcome measures, while 34% reported dissatisfaction. Fourteen respondents elaborated the reason for being unsatisfied, which was mainly related to limited access to quantitative outcome measures that aid in reducing subjective bias and detect accurately the client’s progress.

#### Barriers to assessment

SLTs responded to closed and open-ended questions related to perceived barriers to effective assessment in adults with non-progressive dysarthria. All the respondents’ comments were accounted for, and no responses were ignored. 78% of respondents working at medical centers reported that the centers don’t provide them with the required assessment tools. Out of them, 17 respondents specified the reason behind this; Ten respondents (58.8%) reported that medical centers assign low budgets for purchasing formal and instrumental assessments due to financial problems, while seven respondents (41.2%) asserted that this is related to the unavailability of Arabic formal assessments.

Furthermore, SLTs stated their influences on the choices of assessment tools. The main factor reported to influence respondents’ choice was the availability of the test (60%, *n* = 30), and some designated preference to tests that are free of charge. Others specified that the clinical relevance of the assessment tool (22%, *n* = 11), the ease of use and administration (18%, *n* = 9), and the severity of dysarthria (14%, *n* = 7) were key factors for selecting the required assessment. In an open-ended question, respondents provided qualitative information about the perceived barriers and restrictions affecting the proper assessment of adults with non-progressive dysarthria. Five categories were extracted and summarized in Table 4; they included lack of available standardized Arabic assessment tools, comorbidities, financial problems, professional limitations, and client motivation and compliance.

**Table 4:** Barriers to assessment in adults with non-progressive dysarthria

Barriers to effective assessment	Examples of responses	***n***	%
Lack of available standardized Arabic assessment tools	• Absence of tools adapted to the Lebanese population. • We don’t have a reliable and valid assessment tool in the Arabic language to assess all areas needed (phonation, prosody, respiration...)	34	68
Comorbidities	• The variability in the characteristics of dysarthria along with comorbidities in patients makes it hard to assess effectively. • The presence of comorbidities and especially in cases of dysphagia. • Mostly, the receptive level of the patient influences the assessment process.	11	22
Financial problems	• Financial barriers to providing specialized instrumental assessment tools. • I am unable to purchase standardized English assessments for my bilingual patients due to the current dollar rate crisis.	10	20
Professional limitations	• Perceptual judgments are subjective as the accuracy of the assessment finding depends on the clinician’s expertise in active listening and analyzing the characteristics of speech. • I receive a limited number of patients with dysarthria per year, so, I believe I need more practice.	5	10
Client motivation and compliance	• The low motivation of the patient as the assessment tasks might seem boring.	5	10

### Lebanese SLTs’ attitude towards topics related to the assessment of non-progressive dysarthria

Respondents were asked to state their level of agreement on statements related to the assessment of adults with non-progressive dysarthria (Table 5). In terms of the main reasons behind conducting assessments in adults with non-progressive dysarthria, SLTs (94%) largely agreed with the statement that they “assess people with non-progressive dysarthria to plan therapy”. Most of the SLTs (90%) also agreed with the statement that they “assess people with non-progressive dysarthria to provide feedback and education to client”. However, there was a lack of consensus on whether the assessment of non-progressive dysarthria is performed to establish a differential diagnosis; 48% of SLTs agreed with this statement, while 52% disagreed or stated being neutral.

**Table 5:** The attitude of SLTs towards statements regarding the assessment of adults with non-progressive dysarthria (*n* = 50)

Statement	Agree		Neutral		Disagree	
	***n***	%	***n***	%	***n***	%
I assess people with non-progressive dysarthria to have a differential diagnosis.	24	48	21	42	5	10
I assess people with non-progressive dysarthria to prioritize treatment and/or caseload management.	37	74	10	20	3	6
I assess people with non-progressive dysarthria to inform medical diagnosis.	32	64	10	20	8	16
I assess people with non-progressive dysarthria to provide feedback for service managers.	27	54	15	30	8	16
I assess people with non-progressive dysarthria to evaluate therapy.	40	80	7	14	3	6
I assess people with non-progressive dysarthria to measure the impact of dysarthria on the client’s functional communication.	44	88	3	6	3	6
I assess people with non-progressive dysarthria to establish the wider social impact on the client’s life.	40	80	7	14	3	6
I assess people with non-progressive dysarthria to have information on the potential for improvement.	41	82	6	12	3	6
I assess people with non-progressive dysarthria to measure the severity of dysarthria.	43	86	4	8	3	6
I assess people with non-progressive dysarthria to measure the impact of dysarthria on the client’s participation in society.	40	80	8	16	2	4
I assess people with non-progressive dysarthria to determine baseline information.	40	80	8	16	2	4
I assess people with non-progressive dysarthria to provide feedback for other professionals.	37	74	9	18	4	8
I assess people with non-progressive dysarthria to plan therapy.	47	94	1	2	2	4
I assess people with non-progressive dysarthria to provide feedback and education to the client.	45	90	2	4	3	6
I assess people with progressive dysarthria differently to people with non-progressive dysarthria.	13	26	27	54	10	20
I do not routinely use a formal assessment with this population.	38	76	7	14	5	10
I routinely use the Mayo Classification System in describing the type of dysarthria.	16	32	17	34	17	34
I do not routinely use audio recording as part of my dysarthria assessment.	7	14	7	14	36	72
I do not routinely use video recording as part of my dysarthria assessment.	19	38	10	20	21	42
I find the Mayo classification system useful.	21	42	27	54	2	4
I am confident in my ability to accurately assess people with non-progressive dysarthria.	14	28	28	56	8	16
I am not sure of the value of some of the assessment tools I use.	17	34	24	48	9	18
Currently available assessment tools for non-progressive dysarthria adequately meet my needs.	2	4	16	32	32	64
I need more training in specialized instrumental and acoustic software.	43	86	4	8	3	6

Over 76% of respondents agreed that they use formal assessment with adults with non-progressive dysarthria. However, the SLTs showed a lack of consensus on other statements. 26% of respondents stated that they assess people with progressive dysarthria differently to people with non-progressive dysarthria, although a majority (54%) reported they were not sure. When asked to state agreement with the statement “I routinely use the Mayo Classification System in describing the type of dysarthria”, a similar number of respondents chose “agree”, “neutral” and “disagree”. A further lack of consensus was presented for the statement “I find the Mayo classification system useful”, where 42% of respondents agreed and 54% were not sure. 86% of SLTs agreed that they need more training in specialized instrumental and acoustic software, while 56% reported that they were not surely confident about their ability to accurately assess people with non-progressive dysarthria.

## Discussion

The present study aims to explore the practices and attitudes of Lebanese SLTs who assess adults with non-progressive dysarthria and sites them in a broader context of professional guidelines. In Lebanon, speech and language therapy is considered a new field of specialty, with the first promotion of SLTs being graduated in 1999. Scarce information is available on the assessment practices of Lebanese SLTs, as well as Arab SLTs, in the different clinical areas including non-progressive dysarthria and their adherence to the ICF framework. Collecting information about Arab SLTs’ approaches to assessment and measurement of therapeutic outcomes is highly valuable to ensure that the appropriate tools are being used. Inadequately selected measures fail to accurately evaluate the baseline skills of the client and could not be able to deliver indications for intervention.

The study reported the responses of 50 Lebanese SLTs, nearly half of which reported providing private therapy services at the client’s home. In contrary to studies conducted abroad in the ROI and Australia, SLTs working with adults with non-progressive dysarthria reportedly delivered therapy services primarily at hospitals [16, 17]. It should be remarked that the Lebanese healthcare system is mostly a private sector, where speech and language therapy services are solely delivered in private hospitals or medical centers as well as private clinical settings and are completely funded by the clients and their families. This fact reflects the clients’ preferences for choosing home therapy services that require less waiting time before therapy initiation. Furthermore, SLTs represented variability in years of experience. The diversity in the workplaces and the years of clinical experience did not ascertain any differences in the assessment practices of respondents.

Fortunately, 100% of SLTs reported conducting a form of assessment with clients with non-progressive dysarthria. All SLTs indicated evaluating at least three speech subsystems including articulation, phonation, and respiration. Nevertheless, informal assessments were regularly used by a large proportion of SLTs (86%), while a few numbers of SLTs (14%) used formal assessments; such results correspond to the findings of previous studies [16, 17, 21]. However, this is the case of a distinct reason, where formal or published assessments for non-progressive dysarthria are not available yet in the Arab countries. Furthermore, the qualitative comments of Lebanese SLTs who use formal assessments, standardized in foreign countries, suggested that these measures are either implemented with their bilingual clients or utilized perceptually. This would run in a deviating pattern against the professional recommendations underlining the importance of conducting a detailed assessment without which a differential diagnosis could not be reached, and the therapeutic goals could not be evaluated for effectiveness [33].

Similar to previous studies’ findings [16, 17, 21], the oral motor examination (94%) was the most frequently used informal assessment tool in clients with non-progressive dysarthria, although limited evidence is present on the correspondence of oral motor functions to speech production [34–37]. However, the large proportion of SLTs embedding the oral motor examination within their assessment practices reflects the importance of this tool in evaluating the underlying conditions associated with the client’s initial medical etiology as well as with dysarthria. Although Lebanese SLTs agreed on the importance of assessing communication beyond the level of impairment, responses suggested that they utilize predominantly impairment-based assessments, which align with the findings of previous studies [16, 17, 21]. This may be partly related to the fact that research on the clinical presentation and evaluation of dysarthric symptoms is largely impairment-focused [4, 18]. On the other hand, dysarthria-specific impact and participation measures are present and published [38, 39], however, they are not translated and validated among the Arabic cultures.

Based on the SLTs’ responses, the application of instrumental assessments was markedly rare. Similarly, previous studies exploring the assessment practices of non-progressive dysarthria in the ROI, UK, and Australia demonstrated similar results [16, 17, 21], along with those observed in the assessment of progressive dysarthria [40]. A recent survey conducted in the UK on the use of technology in the assessment and treatment of motor speech disorders indicated that the majority of SLTs used loudness meters and voice recorders to analyze the voice and speech of their clients during the assessment process [41]. According to the qualitative comments of Lebanese SLTs, they stated that the availability of specialized instrumental tools in Lebanon would be of value, as limited access is reported. In addition, most of the SLTs (86%) agreed that they need more training in instrumental assessments. This indicates that the reasons behind the restricted implementation of instrumental assessments are mainly related to limited availability or access as well as constrained expertise.

Lebanese SLTs acknowledged important value for the assessment of functional communication beyond the clinical setting and investigating the underlying barriers. Recent studies have shed light on the psychosocial impact of dysarthria on the client’s life and participation in society [3, 4, 18], which seemed to raise SLTs’ awareness toward these issues and influenced their perspectives on related mental health problems. Generally, the assessment practices of Lebanese SLTs respected the ICF framework, as they tended to define the functional profiles of adults with non-progressive dysarthria. Lebanese SLTs reported the importance of evaluating the interaction between the motor speech condition and the environmental factors of the society in which individuals with non-progressive dysarthria live. However, SLTs relied mostly on informal assessments to evaluate the activity and participation domains of dysarthria, since comprehensive formal assessments targeting these areas are scarce in the Arab countries. These findings were found consistent with previous studies [16, 17, 21], where SLTs favored informal assessments despite the presence of several validated and standardized formal assessments.

Several barriers to effective assessment were documented by Lebanese SLTs. Some of which were consistent with the reported findings of previous studies including clinical comorbidities, professional limitations, and client’s compliance [16, 17, 21]. Additionally, a notable proportion of SLTs complained about financial problems. To elaborate more, 2 years ago, Lebanon has slid into an economic crisis characterized by a significant progressive decline of the Lebanese pound against the American dollar, leading to a dollar shortage in the country. The devaluation of the Lebanese currency severely constrained the purchasing capacity of Lebanese SLTs and limited the funding provided by the medical centers or workplaces. The captured qualitative comments suggested that several SLTs were not able to pay for formal assessments and instrumentations and reported using free assessment tools including free acoustic softwares or apps that are relatively accessible on smartphones or tablets. Also, the qualitative comments supported the fact that most of the SLTs could not afford international specialized trainings which are mostly offered in American dollars. Still, the most-reported barrier to effective assessment was the scarcity of Arabic standardized assessments available for non-progressive dysarthria. Only 4% of respondents reported that they were satisfied with the currently available assessment tools. Furthermore, test availability was reported as a key factor influencing assessment choice. Therefore, the development and validation of Arabic assessments for non-progressive dysarthria are highly recommended and valued as these measures can potentially depict the efficacy of the chosen treatment modalities, guide clinical practice, and thus improve clinical outcomes.

SLTs have the responsibility to maintain competency and up-to-date learning. Lebanese SLTs are facing daunting challenges to enhance professional practices during the current Lebanese crisis. This is related to their limited access to educational resources, e-learning modules, and specialized training programs due to financial concerns. The findings of this study disclose that the assessment practices of Lebanese SLTs working with adults with non-progressive dysarthria follow the international guidelines. Therefore, SLTs are recommended to develop interprofessional collaboration groups to share experiences and resources and thus improve therapeutic outcomes. Such collaborative practices will help in promoting SLTs’ skills and identify the present gaps in their area of practice.

It is important to mention the strengths and limitations of this study. To the best of our knowledge, this is the first study conducted in Lebanon and the Arab world to investigate the assessment practices of SLTs working with non-progressive dysarthria. Research on speech therapy-related topics are extremely limited in the Arab area, and thus, there is a paucity of information on the general practices of Arab SLTs. In sum, the present study can support the development of speech therapy services in Arab countries and hypothesize future research statements. The findings of the present study must be considered in light of several limitations. Firstly, in Lebanon, there is no national register for the profession, so, a response rate couldn’t be estimated; thus, the findings of this study could not be generalized to reflect the assessment practices of all Lebanese SLTs. Secondly, whilst the findings of this study shed light on important and novel findings, a larger sample size could have added more statistical power. When compared with other studies conducted previously in ROI, UK, and Australia [16, 17, 21], the sample size of this study is relatively similar, knowing that Lebanon is a very small country. Thirdly, the survey methodology is limited as it presented specific response options [40], which might not necessarily reflect SLTs’ true perspectives and clinical practices. Fourth, it is possible that SLTs may have been biased to respond to questions about their assessment practices in a way that reflects valued standards more than actual practices. Nonetheless, this manuscript will encourage further studies, of larger sample sizes, to be conducted in the Arab countries to ensure that Arab SLTs are practicing according to prevailing professional standards. On the other hand, the findings provided here support a need for the establishment and standardization of Arabic measures to quantitatively evaluate dysarthria.

## Conclusion

This study is the first initiative in Lebanon and the Arab countries to explore the assessment practices and attitudes of SLTs who work with adults with non-progressive dysarthria. The vast majority of the SLTs perceived the importance of assessing all the ICF domains. However, the different aspects of non-progressive dysarthria were mostly evaluated qualitatively by Lebanese SLTs, in the absence of Arabic standardized tools. SLTs reported lacking the necessary assessments and highlighted the need for valid and reliable measures for non-progressive dysarthria. Fortunately, the assessment practices of Lebanese SLTs don’t differ very much from what previous surveys elsewhere have discovered. The results presented in this study are an important contribution to the clinical field as they provided insight regarding novel information about the profession in Lebanon.

## Supplementary Information

**Additional file 1.**

## Data Availability

Data are available from the corresponding authors upon reasonable request.

## Abbreviations

SLTs: Speech and language therapists
ICF: International classification of functioning, disability and health
ROI: Republic of Ireland
UK: United Kingdom
TBI: Traumatic brain injury
ALO: Association Libanaise des orthophonistes
ISO-USJ: The superior institute of speech therapy at the Saint Joseph university
IBM SPSS: Statistical package for social sciences
SD: Standard deviations
BECD: Batterie d’évaluation clinique de la dysarthrie
FDA: Frenchay dysarthria assessment
RDA: Radboud dysarthria assessment
VHI-10 lb.: The Lebanese arabic version of the voice handicap index-10
