# Supplementary material for: Assessment of non-progressive dysarthria: practice and attitude of speech and language therapists in Lebanon
Source: BMC Neurol. 2021 Nov 17;21:450. doi: 10.1186/s12883-021-02484-2 (PMC8596921; doi:10.1186/s12883-021-02484-2)
Supplement: Supplementary file 1 — Additional file 1. [file 12883_2021_2484_MOESM1_ESM.docx]

**Assessment of non-progressive dysarthria: practice and attitude of speech and language therapists in Lebanon**

Marwa Summaka^a^, Hayat Harati^b^, Salem Hannoun^c^, Hiba Zein^d^, Nour Koubaisy^d^, Youssef Fares^b^ and Zeina Nasser^b^*

^a^Doctoral School of Sciences and Technology, Lebanese University, Hadath, Lebanon; ^b^Faculty of Medical Sciences, Neuroscience Research Center, Lebanese University, Hadath, Lebanon; ^c^Medical Imaging Sciences Program, Division of Health Professions, Faculty of Health Sciences, American University of Beirut, Beirut, Lebanon; ^d^Department of Rehabilitation, Health, Rehabilitation, Integration and Research Center (HRIR), Beirut, Lebanon.

Corresponding author: Zeina Nasser ^a^* MPH, PhD, Faculty of Medical Sciences, Neuroscience Research Center, Lebanese University, Hadath, Lebanon. Email: [z.nasser@ul.edu.lb](mailto:z.nasser@ul.edu.lb); ORCID: Zeinanasser2020; Telephone: 961 1610923-Fax: 961 1610920.

**Online Survey**

# **Demographics**

1. Gender *(Male, Female)*
2. Age
3. Marital status *(Single, Married, Engaged, Divorced, Widowed)*
4. Highest level of education in speech therapy *(Bachelor degree, Masters degree, Ph.D.)*
5. Years of experience in the field of adult non-progressive dysarthria assessment and intervention
6. Primary workplace *(Acute general hospital, Post-acute/rehabilitation general hospital – inpatient, Outpatient hospital, Residential/nursing home, Rehabilitation center, Client’s home, Private clinic, Telepractice)*

# **General information**

1. What portion of your current caseload is spent in working with people with non-progressive dysarthria? *(Less than or about 25%, Less than or about 50%, Less than or about 75%, Greater than 75%)*
2. What is the primary underlying medical condition resulting in non-progressive dysarthria in clients on current caseload? *(Stroke, Traumatic brain injury, Cerebral palsy, Aneurysm, Hypoxia, Encephalitis, other)*
3. Specify the referral sources of your people with non-progressive dysarthria. *(Medical doctors, Therapists from different rehabilitative fields, Client’s/ client’s family decision)*

# **Practices section**

## **Assessment**

**Please answer the following questions about your assessment practices in people with non-progressive dysarthria.**

| 1. Which components of speech production do you assess in people with non-progressive dysarthria? *(Always, Most of the time, Some of the time, Never)* |
| --- |
| 1. Respiration |
| 1. Prosody |
| 1. Resonance |
| 1. Articulation |
| 1. Phonation |
| 1. How often do you use descriptive assessments and informal tools in people with non-progressive dysarthria? *(Always, Most of the time, Some of the time, Never)* |
| 1. How often do you use the following descriptive assessments and informal tools in people with non-progressive dysarthria? *(Always, Most of the time, Some of the time, Never)* |
| 1. Oral facial examination |
| 1. Informal articulation rating |
| 1. Informal speech rating |
| 1. Informal intelligibility testing |
| 1. Conversation/interaction rating |
| 1. Observation of connected speech in reading |
| 1. Informal rating scales of speech function (e.g. breath support and phonation) |
| 1. Client’s/significant others’ ratings of social participation |
| 1. Informal ratings of conversational partner interaction skills |
| 1. Other *(specify)* |
| 1. How often do you use formal assessment tools in people with non-progressive dysarthria? *(Always, Most of the time, Some of the time, Never)* |
| 1. Specify the formal assessment tool used to assess people with non-progressive dysarthria. *(comment)* |
| 1. How often do you use the following instrumentation in your assessment of people with non-progressive dysarthria? *(Always, Most of the time, Some of the time, Never)* |
| 1. Computerized speech lab (CSL) |
| 1. Visispeech |
| 1. Visipitch |
| 1. Acoustic analysis software package (e.g., Praat, lingWAVES) |
| 1. Analysis of Dysphonia in Speech and Voice (ADSV^TM^) |
| 1. Electroglottograph (EGG) |
| 1. Other *(specify)* |
| 1. How often do you assess the following areas in people with non-progressive dysarthria? *(Always, Most of the time, Some of the time, Never)* |
| 1. Communication effectiveness beyond the clinical setting |
| 1. Environmental barriers to communication |
| 1. Attitudinal barriers to communication |
| 1. Depression/anxiety |
| 1. Quality of Life |
| 1. Psychosocial impact of dysarthria |
| 1. How often do you use the Mayo classification system (e.g., flaccid, spastic, unilateral upper motor neuron, etc.) in describing the type of dysarthria? *(Always, Most of the time, Some of the time, Never)* |

**Open questions about assessment practices:**

1. Does the institution or medical center you work at provide you with the assessment tools needed? *(Yes, No; if No specify the reason)*
2. Are there any aspects of speech, communication, interaction, and/or participation that you would like to address in the assessment but do not have the necessary tools to do so? *(Yes, No; if Yes specify the reason)*
3. State influences on choices of assessment tools. *(comment)*
4. In your opinion, what are the barriers to the effective assessment of people with non-progressive dysarthria? *(comment)*

## **Outcome measures**

**Please answer the following questions about your assessment practices in people with non-progressive dysarthria.**

| 1. How often do you implement post-therapy re-assessment? *(Always, Most of the time, Some of the time, Never)* |
| --- |

**Questions about outcome measures:**

1. What outcome measures do you implement to evaluate client outcomes? *(Goal attainment scaling, Repeat formal/published assessment, Repeat instrumental measures, Repeat informal assessments)*
2. What is your reason for using or not using outcome measures? *(comment)*
3. Do these outcome measures meet/fulfill your needs? *(Yes, No)*. If you have reported that the outcome measures you are using do not meet your needs. Please elaborate on why this is the case. *(comment)*

# **Attitude section**

**26. Please state your degree of agreement with the following statements about the assessment of people with non-progressive dysarthria.**

| 1. I assess people with progressive dysarthria differently to people with non-progressive dysarthria. *(Agree, Neutral, Disagree)* |
| --- |
| 1. I do not routinely use a formal assessment with this population. *(Agree, Neutral, Disagree)* |
| 1. I routinely use the Mayo Classification System in describing the type of dysarthria. *(Agree, Neutral, Disagree)* |
| 1. I do not routinely use audio recording as part of my dysarthria assessment. *(Agree, Neutral, Disagree)* |
| 1. I do not routinely use video recording as part of my dysarthria assessment. *(Agree, Neutral, Disagree)* |
| 1. I find the Mayo classification system useful. *(Agree, Neutral, Disagree)* |
| 1. I am confident in my ability to accurately assess people with non-progressive dysarthria. *(Agree, Neutral, Disagree)* |
| 1. I assess people with non-progressive dysarthria to have a differential diagnosis. *(Agree, Neutral, Disagree)* |
| 1. I assess people with non-progressive dysarthria to prioritize treatment and/or caseload management. *(Agree, Neutral, Disagree)* |
| 1. I assess people with non-progressive dysarthria to inform medical diagnosis. *(Agree, Neutral, Disagree)* |
| 1. I assess people with non-progressive dysarthria to provide feedback for service managers. *(Agree, Neutral, Disagree)* |
| 1. I assess people with non-progressive dysarthria to evaluate therapy. *(Agree, Neutral, Disagree)* |
| 1. I assess people with non-progressive dysarthria to measure the impact of dysarthria on the client’s functional communication. *(Agree, Neutral, Disagree)* |
| 1. I assess people with non-progressive dysarthria to establish the wider social impact on the client’s life. *(Agree, Neutral, Disagree)* |
| 1. I assess people with non-progressive dysarthria to have information on the potential for improvement. *(Agree, Neutral, Disagree)* |
| 1. I assess people with non-progressive dysarthria to measure the severity of dysarthria. *(Agree, Neutral, Disagree)* |
| 1. I assess people with non-progressive dysarthria to measure the impact of dysarthria on the client’s participation in society. *(Agree, Neutral, Disagree)* |
| 1. I assess people with non-progressive dysarthria to determine baseline information. *(Agree, Neutral, Disagree)* |
| 1. I assess people with non-progressive dysarthria to provide feedback for other professionals. *(Agree, Neutral, Disagree)* |
| 1. I assess people with non-progressive dysarthria to plan therapy. *(Agree, Neutral, Disagree)* |
| 1. I assess people with non-progressive dysarthria to provide feedback and education to the client. *(Agree, Neutral, Disagree)* |
| 1. I am not sure of the value of some of the assessment tools I use. *(Agree, Neutral, Disagree)* |
| 1. Currently available assessment tools for non-progressive dysarthria adequately meet my needs. *(Agree, Neutral, Disagree)* |
| 1. I need more training in specialized instrumental and acoustic software. *(Agree, Neutral, Disagree)* |
